# Personality traits predict regression of pelvic girdle pain after pregnancy: a longitudinal follow-up study

**DOI:** 10.1186/s12884-021-03759-9

**Published:** 2021-05-04

**Authors:** Tang Xiangsheng, Gong Long, Shi Yingying, An Xiao, Yi Ping, Tan Mingsheng

**Affiliations:** 1 Department of Orthopaedic, China-Japan Friendship Hospital, Peking Union Medical College, Chinese Academy of Medical College, Beijing, 100853 China; 2 Department of Psychology, Hai Nan branch of Chinese PLA General Hospital, Sanya, 572000 Hainan China; 3 Department of Orthopaedic, Hai Nan branch of Chinese PLA General Hospital, Sanya, 572000 Hainan China; 4 Department of Orthopedic, China-Japan Friendship Hospital, No. 2 Yinhuayuan East Street, Chaoyang, Beijing, 100029 China

**Keywords:** Low back pain, Pelvic girdle pain (PGP), Pregnant women, Personality traits, Outcomes

## Abstract

**Background:**

Pelvic girdle pain (PGP) is a multifactorial condition with a partly unknown etiology. This condition can be mentally and physically compromising both during and after pregnancy. To provide all-around preventive measures to improve the recovery from PGP, it is a necessity for obstetricians and orthopaedists to develop predictive studies about the worse prognosis for this condition. Therefore, this study aims to determine whether personality traits can predict the consequences of long-term pregnancy-related PGP.

**Methods:**

This was a prospective study conducted from January 2015 to August 2018. A total of 387 pregnant women were enrolled in this study. According to whether they had experienced PGP during the past 4 weeks, the subjects were classified into no PGP and PGP groups. Persistent PGP after the pregnancy was defined as a recurrent or continuous visual analog score (VAS) pain rating of ≥3 for more than 1 week. The Quick Big Five Personality Test (QBFPT) was used to assess personality traits***.*** Data were obtained by mail or in the clinic. The authors collected data including age, BMI, educational level, annual household income, cesarean delivery, breastfeeding, unexpected sex of the baby, parity, sick leave, no or rare ability to take rest breaks at work, and PGP in the previous pregnancy.

**Results:**

Of 387 included women, 264 subjects experienced PGP during the pregnancy with a mean age of 26.3 ± 4.5 years. A total of 80 of 264 (30.3%) women experienced persistent PGP after the pregnancy. Persistent PGP after the pregnancy was associated with higher levels of neuroticism (OR = 2.12, *P* = 0.001). Comparing women with persistent PGP, those who reported higher levels of extraversion and conscientiousness were more likely to recover from this condition (OR = 0.65, *P* = 0.001; OR = 0.78, *P* = 0.010, respectively). Besides, neuroticism was positively associated with higher pain scores (r = 0.52, *P* = 0.005). However, extraversion and conscientiousness domains showed negative correlations with pain score (*r* = − 0.48, *P* = 0.003; r = − 0.36, *P* = 0.001).

**Conclusions:**

Personality traits were significantly associated with the outcomes of PGP.

## Background

Pelvic girdle pain (PGP) is known as a multifactorial disease without definite etiology. This condition may cause mental and physical damage during and after pregnancy [1–3]. It could be severe enough to interfere with daily life, causing limitations in performance and productivity at work [4]. It can be serious enough to compromise daily life as a result of decreased performance and productivity at work for many pregnant women [4]. PGP seemingly increases the frequency initially and remains constant at a higher level, about 35% throughout pregnancy [5]. After delivery, this condition generally diminishes in week 11 postpartum [6]. However, in some patients, PGP does not regress as expected and even progressed to disability associated with sick leave [2, 6]. The prevalence of PGP from the postpartum stage to 3 years and 6 years after delivery is from 1 to 43% [7] and 7% [8], respectively, in previous studies.

The predictors and long-term outcomes of PGP associated with pregnancy have been studied. Increasing evidence indicates that many factors, such as demographic characteristics (e.g., age, occupation), pregnancy-related condition (e.g., PGP in early pregnancy), and low endurance of back flexors, are all related to the severity and regression of PGP [9–12]. However, these factors could not wholly explain why some pregnant women suffer more severe PGP or fail to recover from this condition.

Pain exists as a complex experience that involves diverse aspects, including sensory, cognitive, and emotional processes [13]. In terms of the perception of painful feelings, susceptibility to pain status, and response to pain treatment, individual differences are significant [14]. Psychological determinants, such as post-operative catastrophic changes, negative emotions, and expectations, has been increasingly attached importance. This may contribute to identifying patients with greater risks for chronic and disabling pain [15–17]. To be specific, negative emotions, mainly depression and anxiety, have been demonstrated to aggravate the visual analog scale (VAS) [18], which is the most common indicator in the evaluation for PGP [5, 6]. Therefore, psychological status has a connection with pain and serves as a reliable predictor for long-term pregnancy-related PGP consequences.

Personality traits are a relatively stable mental profile to fully reflect a person’s psychological status [14, 18]. Interestingly, pain hypersensitivity is related to personality traits [14, 19]. PGP, as a common condition both during and after pregnancy, could face these similar scenarios. Diagnostic guidelines for PGP indicate that the pain occurs between the posterior iliac crest and the gluteal plica, especially near the sacroiliac joint (SIJ), either alone or in combination with the pain in the symphysis [20]. Postpartum PGP may develop from acute to chronic pain, which is a complex process. It is of great necessity to explore why some women experience long-term PGP after childbirth and which women suffer increased risk. The role of personality traits in predicting the long-term PGP associated with pregnancy has never been investigated. To provide all-around preventive measures to improve the recovery from PGP, it is of great necessity for obstetricians and orthopaedists to develop predictive studies about the worse prognosis for this condition. Therefore, the aims of the present research were to (i) determine if personality traits can predict the consequences of long-term pregnancy-related PGP or not; and (ii) compare the prognosis in different personality traits.

## Methods

### Design

This study was designed as a prospective study, which was performed from January 2015 to August 2018.

### Subjects

Subjects who attended the antenatal clinic at a tertiary care hospital gave written consent. Pregnant women who are usually required to register for obstetrics in the 12th week of pregnancy were included. For obstetric reasons, they are examined on 14 scheduled dates throughout the pregnancy. Subjects were divided into no persistent PGP group and persistent PGP group based on if they had persistent PGP with radiation into one or both legs in the past 4 weeks. The pain should be severe enough to interfere with daily activities for more than a day.

The exclusion criteria were as follows: a history of other low back pain (LBP) such as specific LBP and other unspecific LBP (*N* = 25), the disease or substance abuse prior to the pregnancy (*N* = 21), severe diabetes, hypertension, and other diseases during the whole pregnancy (*N* = 9), sustained extreme fatigue (*N* = 1), major negative events related to the pregnancy such as the accident abortion, severe foetal abnormalities, and other possible reasons for perinatal depression (*N* = 17) [4], pregnancy by reproductive treatments (*N* = 2), and loss follow-up and/or incomplete data (*N* = 7). In the end, 387 pregnant women participate in the study.

### Instruments

#### Assessment

Women experiencing PGP during the pregnancy were arranged for a consultation with a multidisciplinary team, including an orthopaedist, obstetrician, and physiotherapist. During the interview, they could confirm the specific type of LPP, the severity of symptoms, individualized treatments, i.e., medical history. If they fulfilled the inclusion and exclusion criteria and cooperated on further tests, they were scheduled to the multidisciplinary team.

A standardized and reliable assessment [21] was performed to determine PGP based on a history of pain provocation in different postures or daily living activities, pelvic pain provocations tests, and repeated flexion and extension movements with a wide range in standing and/or lying [21]. The pelvic pain induction test includes sacral thrust [21], distraction test, compression test, posterior pelvic pain provocation test [22], and the MAT-test [23].

The definition of PGP is based on the European Guide [20]. Pain occurs between the posterior iliac crest and the gluteal fold, especially near the sacroiliac joint, in combination or alone with the symphysis with decreased endurance during sitting, standing, and walking, and in positive clinical diagnostic tests to reproduce PGP. In addition, after repeated lumbar movement, there exists no nerve root syndrome, no repetitive pain, and/or symptom changes.

**Fig. 1:**
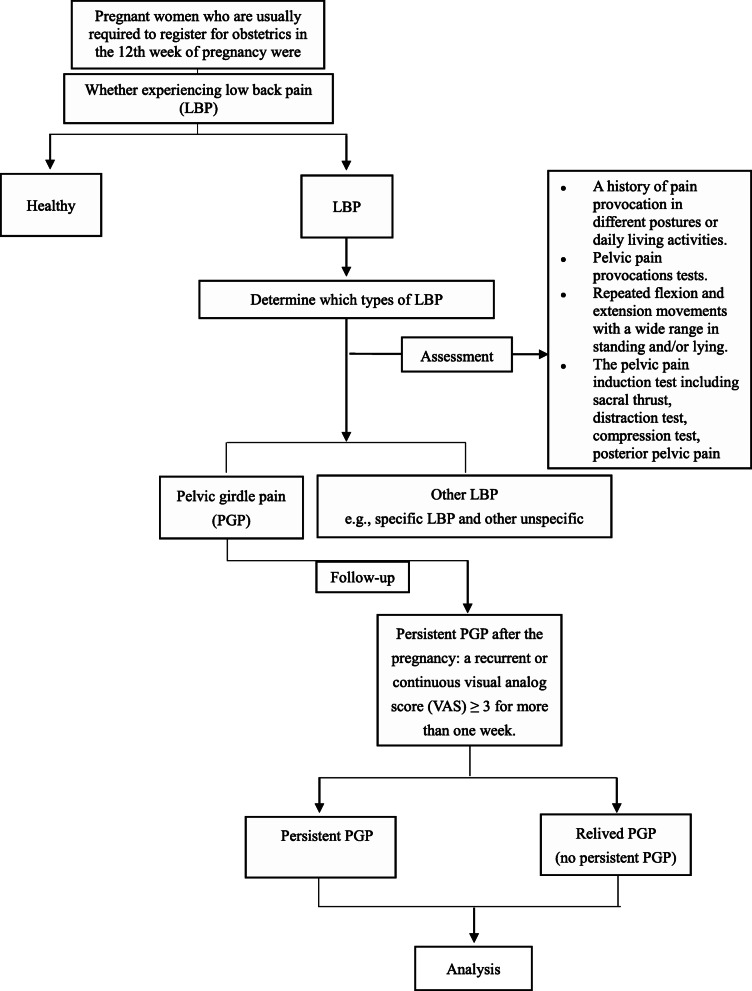
Flow chart for the included women

Persistent PGP after pregnancy is defined as a recurrent or persistent pain score ≥ 3 over a week around 6 months postpartum, as the previous research has shown that persistent PGP generally was significantly improved at this time point [24] and another study indicated that recurrent or persistent lumbar pain score ≥ 3 have been disabled to affect the quality of life [25]. Women with this condition were asked to have a check around 6 months postpartum and 2 years postpartum. But the data “2 years” was used for the final analysis. Pain intensity was assessed using a self-reported scale with a range of 0–10 (0 for no pain, 10 for most pain), which was screened for PGP by interview or phone 2 years after delivery. The flowchart of the included participants can be seen in Fig. 1.

#### Quick big five personality test (QBFPT)

Personality traits were assessed using the QBFPT developed by Vermulst and Gerris (2005) [26] in the 12th week of pregnancy when the subjects were included. This five-trait personality measures include agreeableness (interpersonal trust and thoughtfulness), extraversion (sociability and high activity), conscientiousness (determination, diligence, and organization), neuroticism (distress, usableness to control urgency and deal with pressure, and unrealistic ideas), and openness to experience (aesthetic, sympathy diversity and intellectual curiosity). The measure is a 7-point Likert scale from “completely wrong” (1) to “completely right” (7). Thus, scores on each subscale ranged from 6 to 42. The personality types of individuals are not determined according to a certain range of points. Instead, the score of a participant represented a total score from the high and low scores obtained from each category. Cronbach’s alpha of each subscale was as follows: 0.86 for conscientiousness, 0.78 for neuroticism, 0.80 for agreeableness, 0.81 for extraversion, and 0.73 for openness [26].

#### Data about the pregnancy

Data about the pregnancy were obtained by mail or in the clinic. The authors collected data including age, body mass index (BMI), educational background, annual household income, cesarean delivery, breastfeeding, unexpected sex of the baby, parity, sick leave, no or rare ability to take rest breaks at work, and PGP in the previous pregnancy. In the authors’ country, the baby’s sex is an important reason for the feelings of the mother and family members. Previous studies have shown that women who live in cultures where greater value is placed on sons are more likely to suffer from depression if they give birth to a girl [27, 28]. Therefore, we investigate this important confounder in the present study. These data were completed by the subjects prior to the first evaluation. According to the rule that each variable in the analysis has at least 10 events, the number of variables was required to be limited [29].

### Sample size

Using G Power 3.1.9.2, the study power was calculated for the effect size of 0.3, error of the first type 0.05, and the total number of patients with the number of 75. The calculated study power equals 96.23%, which indicates good study power.

### Statistical analysis

Ordinal variables were showed as proportions. Mean and standard deviation, or median and half-quartile ranges, respectively, were used to represent normally and non-normally distributed variables. Continuous variables and dichotomous variables were tested by the Student t-test and chi-square test, respectively. Kruskal-Wallis test was performed to compare nonparametric data at the ordinal level. Pearson correlation coefficient (coefficient, R) was used to test the correlation between the average score in the personality domain and VAS pain scores by controlling the parameter with a correlation value greater than 0.5. A stepwise multivariate logistic regression was used to detect the independent predictors of PGP after the univariate step of all significant variables with *P* < 0.10 as the prerequisite for this stepwise model. Logistic regression analysis was performed to estimate odds ratios (OR) and 95% confidence intervals (CI) to determine the occurrence of PGP for each personality trait. After adjusting the confounding factors, the multivariable logistic model was established through the stepwise elimination of variables of interest in univariate analysis. *P*-values = 0.05 andβ = 0.8 were defined as the statistical significance and power analysis, respectively. SPSS version 22 (SPSS; Chicago, IL, USA) was applied in this study.

## Results

Of 387 included women, 264 subjects experienced PGP during the pregnancy, with the mean age of 26.3 ± 4.5 years. A total of 80 of 264 (30.3%) women experienced persistent PGP 2 years after the pregnancy.

### The comparisons about the characteristics between PGP and no PGP

Table 1 shows the study population’s baseline characteristics by persistent PGP and no persistent PGP after the pregnancy. More women with persistent PGP after the pregnancy had PGP in the previous pregnancy (55.0% vs. 26.1%, *P* < 0.001) and no or rare ability to take rest breaks at work (37.5% vs. 14.7%, *P* < 0.001) in comparison with those with no persistent PGP during follow-up.

**Table 1:** The comparisons about the baseline characteristics between persistent PGP and no PGP after the pregnancy

	No Persistent PGP (*n* = 184)	Persistent PGP (*n* = 80)	*P* value
Age (Mean ± SD) (years)	26.2 ± 4.3	26.5 ± 4.7	0.613
BMI before pregnancy (Mean ± SD) (kg/m2)	23.2 ± 2.0	22.5 ± 1.8	0.076
Educational Levels (≥high school/university) (N,%)	132 (71.7%)	55 (68.8%)	0.731
Household annual income (Dollars)	1422.5 ± 700.3	1512.5 ± 720.3	0.342
Caesarean delivery (N, %)	26 (14.1%)	11 (13.8%)	0.912
Breast-feeding (N, %)	153 (83.2%)	68 (85.0%)	0.848
Primigravida (N, %)	136 (73.9%)	56 (70.0%)	0.613
Birthweight last born baby, grams (Mean ± SD)	3463 (462)	3512 (470)	0.4315
Sex of last-born baby, boy (N, %)	93 (50.5%)	42 (52.5%)	0.874
No or rare ability to take rest breaks at work (N, %)	27 (14.7%)	30 (37.5%)	< 0.001*
PGP in previous pregnancy (N, %)	48 (26.1%)	44 (55.0%)	< 0.001*

*P* values from t-test or Chi test. *indicates statistically significant. *PGP* Posterior Gridle Pain, *LBP* Low back pain. Combined Pain, *SD* Standard Deviation, *BMI* Body Mass Index

### The comparisons about the median of the subscales of the QBFPT scores between PGP and no PGP

Table 2 shows the subscales of neuroticism (*P* < 0.001) had a negative effect on the recovery from persistent PGP. In contrast, the subscales of extraversion (*P* = 0.003) and conscientiousness (*P* = 0.001) positively affected the recovery from this condition.

**Table 2:** The comparisons about the median of the subscales of the QBFPT scores between PGP and no PGP

QBFPT (Mean ± SD)	No Persistent PGP (*n* = 184)	Persistent PGP (*n* = 80)	*P* value
Agreeableness	30.8 ± 5.3	31.3 ± 5.4	0.484
Extraversion	28.5 ± 6.0	26.1 ± 6.2	0.003*
Conscientiousness	25.2 ± 4.5	23.2 ± 4.8	0.001*
Neuroticism	22.0 ± 6.0	24.0 ± 6.2	< 0.001*
Openness to experience	26.7 ± 5.0	25.9 ± 6.0	0.263

*P* values from t-test. *indicates statistically significant. *PGP* Posterior Gridle Pain, *LBP* Low back pain. Combined Pain

### Logistic regression analyses for persistent PGP after the pregnancy

Table 3 shows the logistic regression analysis results using persistent PGP after the pregnancy as a dependent variable. Persistent PGP after the pregnancy was associated with higher levels of neuroticism (OR = 2.12, *P* = 0.001). Compared to persistent PGP after the pregnancy, women who reported higher levels of extraversion and conscientiousness were more likely to recovery from this condition (OR = 0.65, P = 0.001; OR = 0.78, *P* = 0.010, respectively).

**Table 3:** Associations between women’s personality and persistent pelvic girdle pain (PGP)

Domains	Persistent PGP after the pregnancy			
	Unadjusted		Adjusted^a^	
	OR (95%CI)	*p* value	OR (95%CI)	*p* value
Agreeableness	0.82 (0.78–0.86)	0.720	0.88 (0.83–0.93)	0.626
Extraversion	0.65 (0.62–0.68)	0.005*	0.79 (0.71–0.87)	0.004*
Conscientiousness	0.78 (0.74–0.82)	0.010*	0.92 (0.87–0.97)	0.021*
Neuroticism	2.12 (2.01–2.23)	0.001*	2.03 (1.92–2.13)	0.002*
Openness to experience	1.39 (1.32–1.46)	0.762	1.23 (1.12–1.34)	0.928

*OR* Odds ratio, *CI* Confidence interval. ^a^means adjusted for demographic variables (income, parity, maternal age, education, no or rare ability to take rest breaks at work and PGP in previous pregnancy. *indicates statistically significant

These associations remained significant after adjusting for demographic variables (see Table 3).

### Correlation analyses between mean scores in personality domains and VAS pain score

As shown in Table 4, the correlation analysis results demonstrated that neuroticism was positively correlated with pain scores (*r* = 0.52, *P* = 0.005). However, extraversion and conscientiousness domains revealed negative associations with pain score (*r* = − 0.48, *P* = 0.003; *r* = − 0.36, *P* = 0.001).

**Table 4:** Correlation between mean scores in personality domains and VAS pain score

Domains	Univariable		
	Pearson correlation coefficient (r)	R^**2**^	***p*** value
Agreeableness	−0.23	0.05	0.235
Extraversion	−0.34	0.12	0.003*
Conscientiousness	0.12	0.01	0.001*
Neuroticism	0.39	0.15	0.005*
Openness to experience	0.10	0.01	0.253

*indicates statistically significant

## Discussion

Personality refers to individual differences in characteristic patterns of behaving, feeling, and thinking [26]. There have been several cross-sectional and longitudinal studies investigating the relationship between personality and various health behaviors in the general population. The present research examined the correlations between personality and persistent PGP after the pregnancy by analyzing a sample of 264 (30.3%) women with a two-year follow-up period. Lower extraversion and conscientiousness, and higher neuroticism, were demonstrated to be correlated with continuous PGP for up to 2 years. In present cohorts, higher neuroticism and lower conscientiousness generally have a close association with more intensive pain levels, and increasing evidence of this association has been revealed in non-clinical samples [30, 31]. There is also some evidence that extraversion has a close connection with decreased pain feelings [32, 33]. Similarly, the current study adds that personality could prospectively predict who would bear more risks for sustained PGP in the 2 years after the childbirth.

Both physical and psychological factors could strength the correlations between pain scores and personality traits. Patients with chronic disease, for instance, generally tend to suffer from tremendous pain and stress [29, 30]. The negative feeling is also considered a risk factor for immense pain over time [32]. Patients lower in conscientiousness and extraversion and higher in neuroticism may have a more significant burden on chronic disease [33–36].

Based on this, there are at least three reasons why personality traits could lead to an increased risk of sustained PGP. First, enhanced sensitivity to pain feeling exists in some personality traits. A person would report different pain intensity even if the stimulus is constant [37]. Those who tended to be neurotic could show increased sensitivity for experiencing pain, which thus makes them suffer more from it. One experimental study showed that highly neurotic persons reported more pain than those who were less neurotic under the same laboratory-induced stimuli [38]. In another pain experiment, neuroticism was associated with more intense pain, which sustained a week after the stimulation [39]. The findings suggest it is more likely for neurotic individuals to have a less easily diminished experience of pain feelings over time.

Second, emotional stability is manifested as a particular subject’s tendency towards negative emotion, depression, and anxiety. Extraversion and neuroticism are demonstrated as the two emotional traits most closely connected with negative feelings. Subjects who are more reactive and less emotionally stable are more likely to have adverse reactions. A tendency to be emotionally unstable is common in those who are highly neurotic [39]. Studies have indicated that mothers with low scores in emotional stability are more likely to prefer a cesarean delivery and have complications during delivery, including failure to progress, foetal distress, and severe tearing [38, 40]. The present results further find that high neuroticism relates to persistent PGP. The extraversion is inclined not to be associated with pain sensitivity in response to a stimulus [38, 39]. Extraversion tends to affirmative emotions, sociability, and high activity. These characteristics could give impetus to join sports clubs such as swimming, yoga and be more willing to share experience about PGP’s alleviation method, which has been demonstrated to help recovery from chronic pain [35, 39]. Third, personality affects health-related behaviors, which could be related to pain.

Strong evidence has been found that conscientiousness, extraversion, and neuroticism have connections with some behaviors that make the risk of experiencing pain arise [37, 40, 41]. For instance, patients high in conscientiousness, responsible, self-disciplined, and inclined to adhere to social norms consume less alcohol and fewer cigarettes than average [40, 41]. Higher neuroticism and lower extraversion tend to have physical inactivity [41], poor sleep patterns [40], and tobacco use [42]. These behavioral factors have also been demonstrated to aggravate pain [40–42].

Perinatal depression is a common mental disorder in pregnancy and lactation, with a prevalence between 8 and 36% around the world. This condition could threaten the health of pregnant women and even children [43]. In order to diminish the influence of depression as a confounder for the PGP [44], patients who had many psychological, psychosocial, socioeconomic, and obstetric risk factors reported to be connected with this mental disorder in previous studies [42–44] were excluded as soon as possible (see exclusion criteria mentioned above).

These findings showed that personality traits affect the intensity of PGP and its recovery, and thus could be applied in clinical settings following the replication of these results. Women with high neuroticism should be observed for signs of PGP and high dissatisfaction with the quality of life, and be given preventive interventions such as lumbodorsal muscles and having a rest. What’s more, women with high neuroticism and persistent PGP might benefit from treatments that have been demonstrated to decrease neuroticism and increase extraversion, such as cognitive-behavioral therapy [43]. Also, it might be helpful for those women with negative affect and maladaptive response to pain feeling to accept the metacognitive and insight therapies [45]. Personality scales such as QBFPT and Eysenck Personality Questionnaire are reliable, feasible, and valid in clinical practice. Adding them to the antenatal assessment of women with persistent PGP and neuroticism might allow for better preventive measures, treatment planning, and prediction of prognosis.

The current study included several advantages, such as a measurement of five personality traits, a relatively prospectively long-term assessment of pain, and a focus on pregnant women suffering PGP. These complement the vacancy related to pregnancy-related LBP in previous research. To our knowledge, this is the first study that investigated the associations between personality traits and pregnant women with continued PGP. There are some clinical implications. This study helps the practitioner identify who is most at risk for persistent PGP. Specifically, the present results revealed that individual differences in psychological dispositions are closely related to PGP and its prognosis. Such findings may also be useful for interventions. For instance, interventions themselves might depend on the individual’s personality. Despite not demonstrated in the present study, personality-based interventions have been used smoothly in other fields, such as prevention programs for adolescent alcohol use and misuse [46] and improving behavioral symptoms of dementia [47].

There were several limitations to our study. First, the confounding factor could compromise the reliability of the results, particularly for the potential risk factors for perinatal depression. The etiology of pregnancy-related depression is multifactorial and complex. Despite trying to control its potential risk factors as much as possible, it is unrealistic to eliminate all the adverse events and their effect on each subject’s life. Second, we didn’t adjust the *P*-value for multiple comparisons. While adjusting *p* values contributes to minimizing the Type I errors, such adjustments can be overly conservative and increase the Type II errors. Last, our assessment of maternal personality traits was completed using a standard tool, the QBFPT, developed by Vermulst and Gerris. However, there are several other measures in which they have a specific difference in descriptions about the personality traits. A future study about comparing the reliability and accuracy among them and providing preventive strategies to reduce the severity of PGP during the pregnancy and improve its worse recovery after pregnancy is needed. These limitations bring up the necessity of further studies.

## Conclusions

Persistent PGP after the pregnancy was associated with higher levels of neuroticism. Comparing women with persistent PGP, those who reported higher levels of extraversion and conscientiousness were more likely to recover from this condition. Besides, neuroticism was positively associated with higher pain scores. However, extraversion and conscientiousness domains showed negative correlations with pain score. Personality traits were significantly associated with the outcomes of PGP. These findings showed that personality traits affect the intensity of PGP and its recovery, and thus could be applied in clinical settings following the replication of these results.

## Data Availability

The datasets generated and/or analyzed during the current study are not publicly available due to restrictions associated with the anonymity of participants but are available from the corresponding author on reasonable request.

## Abbreviations

PGP: Pelvic girdle pain
VAS: Visual analogy score
QBFPT: Quick Big Five Personality Test
OR: Odds ratio
IRB: Institutional Review Board
LBP: Low back pain
SD: Standard Deviation
BMI: Body mass index
CI: Confidence interval

## References

[1] Kanakaris NK, Roberts C, Giannoudis P (2011). Pregnancy-related pelvic girdle pain: an update. BMC Med.

[2] Bergstrom C, Persson M, Mogren I (2014). Pregnancy-related low back pain and pelvic girdle pain approximately 14 months after pregnancy - pain status, self-rated health and family situation. BMC Pregnancy Childbirth.

[3] Gutke A, Lundberg M, Ostgaard HC, Oberg B (2011). Impact of postpartum lumbopelvic pain on disability, pain intensity, health-related quality of life, activity level, kinesiophobia, and depressive symptoms. Eur Spine J.

[4] Gutke A, Betten C, Degerskär K, Pousette S, Olsén MF (2015). Treatments for pregnancy-related lumbopelvic pain: a systematic review of physiotherapy modalities. Acta Obstet Gynecol Scand.

[5] Ostgaard HC, Zetherstrom G, Roos-Hansson E (1994). Reduction of back and posterior pelvic pain in relation to pregnancy. Spine (Phila Pa 1976).

[6] Ostgaard HC (1996). Assessment and treatment of low back pain in working pregnant women. Semin Perinatol.

[7] Mogren IM (2006). BMI, pain and hyper-mobility are determinants of long-term outcome for women with low back pain and pelvic pain during pregnancy. Eur Spine J.

[8] Ostgaard HC, Zetherstrom G, Roos-Hansson E (1997). Back pain in relation to pregnancy: a 6-year follow-up. Spine (Phila Pa 1976).

[9] Elden H, Gutke A, Kjellby-Wendt G, Fagevik-Olsen M, Ostgaard HC (2016). Predictors and consequences of long-term pregnancy-related pelvic girdle pain: a longitudinal follow-up study. BMC Musculoskelet Disord.

[10] Munro A, George RB, Chorney J, Snelgrove-Clarke E, Rosen NO (2017). Prevalence and predictors of chronic pain in pregnancy and postpartum. J Obstet Gynaecol Can.

[11] Malmqvist S, Kjaermann I, Andersen K, Gausel AM, Økland I, Larsen JP, Bronnick KS (2018). Can a bothersome course of pelvic pain from mid-pregnancy to birth be predicted? A Norwegian prospective longitudinal SMS-track study. BMJ Open.

[12] Gutke A, Ostgaard HC, Oberg B (2008). Predicting persistent pregnancy-related low back pain. Spine (Phila Pa 1976).

[13] Moayedi M, Davis KD (2013). Theories of pain: from specificity to gate control. J Neurophysiol.

[14] Pud D, Eisenberg E, Sprecher E, Rogowski Z, Yarnitsky D (2004). The tridimensional personality theory and pain: harm avoidance and reward dependence traits correlate with pain perception in healthy volunteers. Eur J Pain.

[15] Badura-Brzoza K, Zajac P, Kasperska-Zajac A (2009). Psychological and psychiatric factors related to quality of life after total hip replacement: preliminary report. Eur Psychiatry.

[16] Riediger W, Doering S, Krismer M (2010). Depression and somatisation influence the outcome of total hip replacement. Int Orthop.

[17] Mannion AF, Kampfen S, Munzinger U, Kramers-de QI (2009). The role of patient expectations in predicting outcome after total knee arthroplasty. Arthritis Res Ther.

[18] Pud D, Yarnitsky D, Sprecher E, Rogowski Z, Adler R, Eisenberg E (2006). Can personality traits and gender predict the response to morphine? An experimental cold pain study. Eur J Pain.

[19] Sjodahl J, Gutke A, Oberg B (2013). Predictors for long-term disability in women with persistent postpartum pelvic girdle pain. Eur Spine J.

[20] Vleeming A, Albert HB, Ostgaard HC, Sturesson B, Stage B (2008). European guidelines for the diagnosis and treatment of pelvic girdle pain. Eur Spine J.

[21] Gutke A, Kjellby-Wendt G, Oberg B (2010). The inter-rater reliability of a standardised classification system for pregnancy-related lumbopelvic pain. Man Ther.

[22] Ostgaard HC, Zetherstrom G, Roos-Hansson E (1994). The posterior pelvic pain provocation test in pregnant women. Eur Spine J.

[23] Fagevik Olsen M, Elden H, Gutke A (2014). Evaluation of self-administered tests for pelvic girdle pain in pregnancy. BMC Musculoskelet Disord.

[24] Ostgaard HC, Roos-Hansson E, Zetherstrom G (1996). Regression of back and posterior pelvic pain after pregnancy. Spine (Phila Pa 1976).

[25] Verkerk K, Luijsterburg PA, Miedema HS, Pool-Goudzwaard A, Koes BW (2012). Prognostic factors for recovery in chronic nonspecific low back pain: a systematic review. Phys Ther.

[26] Vermulst AA, Gerris JRM (2005). QBF: Quick Big Five Persoonlijkheidstest Handleiding.

[27] Hu Y, Wang Y, Wen S, Guo X, Xu L, Chen B, Chen P, Xu X, Wang Y (2019). Association between social and family support and antenatal depression: a hospital-based study in Chengdu, China. BMC Pregnancy Childbirth.

[28] Senturk Cankorur V, Duman B, Taylor C, Stewart R (2017). Gender preference and perinatal depression in Turkey: a cohort study. PLoS One.

[29] Peduzzi P, Concato J, Kemper E, Holford TR, Feinstein AR (1996). A simulation study of the number of events per variable in logistic regression analysis. J Clin Epidemiol.

[30] Bucourt E, Martaillé V, Mulleman D, Goupille P, Juncker-Vannier I, Huttenberger B (2017). Comparison of the big five personality traits in fibromyalgia and other rheumatic diseases. Joint Bone Spine.

[31] Cheng H, Furnham A (2013). Factors influencing adult physical health after controlling for current health conditions: evidence from a British cohort. PLoS One.

[32] Bjelland EK, Stuge B, Engdahl B, Eberhard-Gran M (2013). The effect of emotional distress on persistent pelvic girdle pain after delivery: a longitudinal population study. BJOG..

[33] Chang MC, Chen PF, Lung FW (2017). Personality disparity in chronic regional and widespread pain. Psychiatry Res.

[34] Sutin AR, Zonderman AB, Ferrucci L, Terracciano A (2013). Personality traits and chronic disease: implications for adult personality development. J Gerontol B Psychol Sci Soc Sci.

[35] Weston SJ, Hill PL, Jackson JJ (2015). Personality traits predict the onset of disease. Soc Psychol Personal Sci.

[36] Soto CJ (2015). Is happiness good for your personality? Concurrent and prospective relations of the big five with subjective well-being. J Pers.

[37] Sutin AR, Stephan Y, Luchetti M, Terracciano A (2019). The prospective association between personality traits and persistent pain and opioid medication use. J Psychosom Res.

[38] Quan X, Fong DYT, Leung AYM, Liao Q, Ruscheweyh R, Chau PH (2018). Validation of the mandarin Chinese version of the pain sensitivity questionnaire. Pain Pract.

[39] Pallegama RW, Ariyasinghe S, Perera ED, Treede RD (2017). Influence of catastrophizing and personality traits on recalled ratings of acute pain experience in healthy young adults. Pain Med.

[40] Stephan Y, Sutin AR, Bayard S, Križan Z, Terracciano A (2018). Personality and sleep quality: evidence from four prospective studies. Health Psychol.

[41] Sutin AR, Stephan Y, Luchetti M, Artese A, Oshio A, Terracciano A (2016). The five-factor model of personality and physical inactivity: a meta-analysis of 16 samples. J Res Pers.

[42] Cheng H, Furnham A (2016). The big-five personality traits, maternal smoking during pregnancy, and educational qualifications as predictors of tobacco use in a nationally representative sample. PLoS One.

[43] Paschetta E, Berrisford G, Coccia F, Whitmore J, Wood AG, Pretlove S (2014). Perinatal psychiatric disorders: an overview. Am J Obstet Gynecol.

[44] Vergara R, Maher C, Van Kessel G (2018). The comorbidity of low back pelvic pain and risk of depression and anxiety in pregnancy in primiparous women. BMC Pregnancy Childbirth.

[45] Philipp R, Kriston L, Lanio J, Kühne F, Härter M, Moritz S, Meister R (2019). Effectiveness of metacognitive interventions for mental disorders in adults-a systematic review and meta-analysis (METACOG). Clin Psychol Psychother.

[46] Conrod PJ, O’Leary-Barrett M, Newton N, Topper L, Castellanos-Ryan N, Mackie C (2013). Effectiveness of a selective, personality-targeted prevention program for adolescent alcohol use and misuse: a cluster randomized controlled trial. AMA Psychiatry.

[47] Kolanowski A, Litaker M, Buettner L, Moeller J, Costa PT (2011). A randomized clinical trial of theory-based activities for the behavioral symptoms of dementia in nursing home residents. J Am Geriatr Soc.

